# Assessing the Health Threat of Outdoor Air: Lung Cancer Risk of Particulate Matter Exposure

**DOI:** 10.1289/ehp.122-A252

**Published:** 2014-09-01

**Authors:** Julia R. Barrett

**Affiliations:** Julia R. Barrett, MS, ELS, a Madison, WI–based science writer and editor, has written for *EHP* since 1996. She is a member of the National Association of Science Writers and the Board of Editors in the Life Sciences.

Outdoor air pollution is made up of particulate matter (PM) and hundreds of chemicals from natural sources and human-related activities.^1^^,^^2^ In October 2013 the International Agency for Research on Cancer (IARC) reviewed more than 1,000 research articles and formally designated outdoor air pollution in general and PM in particular as human carcinogens.^3^ A new meta-analysis of epidemiological research in this issue of *EHP* now estimates the lung cancer risk associated with PM exposure.^4^

PM includes particles of varying sizes, with the coarse (PM_10_) and fine (PM_2.5_) fractions attracting the most research attention.^4^ PM_2.5_ is of special interest because its size allows delivery of genotoxic chemicals deep into the lung.^3^^,^^5^ Worldwide, average outdoor air concentrations of PM vary from less than 10 μg/m^3^ to more than 100 μg/m^3^, but most studies have been undertaken in North America and Europe, which have relatively low PM levels (10–30 μg/m^3^) compared with those in developing countries.^1^^,^^2^

**Figure:**
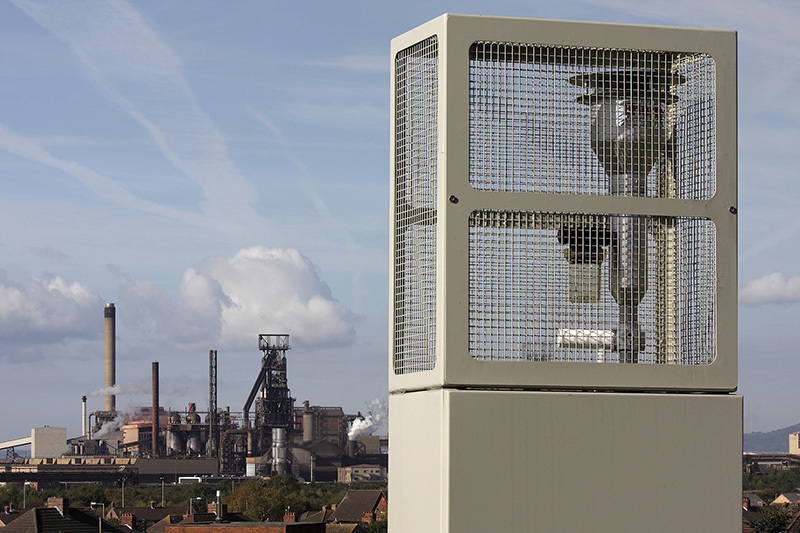
The authors of the new review conducted subgroup analyses to see if it mattered whether PM exposure estimates depended on fixed-site monitors versus modeling techniques. It didn’t; estimated associations between exposure and lung cancer were relatively consistent regardless of the method used to estimate individual exposures. © Jon Bower/Loop Images/Corbis

The meta-analysis focused on 18 large epidemiological studies that estimated residential exposure to PM_2.5_, PM_10_, or both, along with differences in lung cancer incidence or mortality associated with increased exposure. All studies controlled for age and sex, with adjustment for other confounders varying across studies.

The authors of the current review estimated that each 10-μg/m^3^ increase in PM_2.5_ exposure was associated with a 9% increase in lung cancer risk. The estimated risk associated with a 10-μg/m^3^ increase in PM_10_ exposure was similar (8%), but the estimate was less precise.^4^

“Our review assumes that as exposure to PM increases, so does lung cancer risk,” says lead author Ghassan Hamra, now an assistant professor of environmental and occupational health at Drexel University, who conducted the work as a postdoctoral fellow at IARC. “So, rapidly industrializing countries may see an increase in lung cancer incidence. However, it is unclear at what levels of exposure to outdoor air pollution we could expect to see a leveling off of lung cancer risk.”

Subgroup analyses were conducted by continent, PM exposure assessment method (fixed-site monitoring versus model-based estimation), and smoking status (never, former, or current). Continent and assessment method did not alter the overall conclusion. The estimated relative risk for lung cancer in association with PM_2.5_ differed according to smoking status, with current smokers having the weakest association, former smokers the strongest, and never smokers in between. However, the subgroup-specific estimates were imprecise, and the differences were not statistically significant. The authors could not perform a similar analysis for PM_10_ because of a lack of information on patterns of former smoking.^4^

“I think an important direction for future research will be attempting to evaluate the carcinogenicity of components of PM_2.5_,” says Hamra. “Some of these components may be harmful, while others may be innocuous. Further research could help us better understand if this is the case.”

“It’s a very straightforward paper, a very straightforward analysis, applying very well-known, standard techniques,” says Michael Brauer, a professor at the University of British Columbia School of Population and Public Health, who contributed to the IARC evaluation. “The goal of the IARC evaluation is to provide a yes/no answer [as to whether air pollution causes cancer]; it doesn’t provide any quantitative risk estimate. The yes/no answer is helpful, but many people want to go beyond that,” he says.

According to IARC, 223,000 lung cancer deaths due to air pollution occurred worldwide in 2010.^6^ In terms of the global burden of all diseases attributable to air pollution, lung cancer accounts for less than 7% of the 3.22 million estimated deaths.^4^

Still, that risk may provide a strong impetus toward enacting policies and practices for protecting and improving air quality.^3^ “If we think about all the ways air pollution can cause death and disability, the cancer component is actually rather small,” Brauer says. But the idea that air can be carcinogenic is, he says, “very powerful.”
